# School Bag Design and Weight: A Narrative Review of Their Impact on Children's Posture and Musculoskeletal Health

**DOI:** 10.7759/cureus.99595

**Published:** 2025-12-19

**Authors:** Vanshika Singh, Vineet Kumar, Arvind Kumar Singh, Neha Rai, Swagat Mahapatra

**Affiliations:** 1 Department of Orthopaedics, Dr. Ram Manohar Lohia Institute of Medical Sciences, Lucknow, IND; 2 Department of Community Medicine, Dr. Ram Manohar Lohia Institute of Medical Sciences, Lucknow, IND; 3 Department of Pediatrics, Dr. Ram Manohar Lohia Institute of Medical Sciences, Lucknow, IND

**Keywords:** children's posture, ergonomics, musculoskeletal health, school bag design, weight distribution

## Abstract

In recent years, the issue of school bag design and its impact on children's posture has garnered significant attention among educators, parents, and health professionals alike. As students are required to carry increasingly heavy loads of educational materials, the design and weight of school bags can profoundly affect their physical well-being. Improperly designed bags, often exceeding recommended weight limits, can lead to poor posture, spinal misalignment, and long-term musculoskeletal issues. Furthermore, as school-age children are still developing, the physical stresses associated with poorly designed or overloaded bags exacerbate their vulnerability to posture-related problems. This study aims to examine the relationship between various aspects of school bag design, such as ergonomics, weight distribution, and overall functionality, and the posture of school children as reported by their parents. By highlighting these associations, the research seeks to inform guidelines for improving school bag designs to promote healthier postural habits in children.

## Introduction and background

In today's educational environment, school children are often burdened with heavy backpacks that can significantly impact their posture and overall health. Postural issues in children can arise from various factors, one of which is the weight and design of school bags. Poor posture, particularly among school-aged children, has been linked to long-term musculoskeletal issues. With the trend of increasing curricular demands, children often carry heavy loads that may exceed the recommended weight limits, leading to discomfort and potential health problems.

Carrying heavy loads during childhood, when musculoskeletal systems are still developing, significantly raises the risk of chronic postural problems. Therefore, ergonomic bag designs that properly distribute weight are crucial for both immediate comfort and preventing lasting health issues [1]. The physical well-being of school children has emerged as a concern, particularly regarding the design and weight of school bags. Research indicates that the inappropriate use of bags can lead to a range of postural issues among students, adversely affecting their overall health and academic performance. This study seeks to explore the intricate associations between school bag characteristics, such as weight, ergonomic design, and usability, and parent-reported postural habits, and the pivotal role that both parents and teachers play in shaping students' awareness of proper posture and responsible bag usage. Ultimately, a comprehensive understanding of these factors is essential for promoting an environment conducive to both learning and physical health.

## Review

Overview of the importance of school bag design and its impact on children's posture

The weight of school bags may be responsible in influencing children's posture, a factor that is increasingly significant in educational discussions. Properly designed backpacks can reduce the structural stress that school children often experience due to poor weight distribution and inadequate support. A well-structured school bag with ergonomic features promotes an upright posture, reducing the risk of musculoskeletal issues as children grow. In their daily school life, children spend considerable time carrying bags that often exceed recommended weight limits; this can exacerbate poor posture tendencies over time. A study suggests that the prevalence of musculoskeletal pain related to backpack usage among school-going children is high. School children who carry backpacks that weigh more than 15% of their body weight are at risk of experiencing musculoskeletal pain [2]. Furthermore, parental involvement in understanding the implications of bag design is crucial, as they can guide children in selecting appropriate backpacks and educate them on how to wear them correctly [3]. Fostering awareness about the importance of bag design is essential for maintaining children's physical health and ensuring their academic performance is not hindered by preventable posture-related issues [4].

Bag policy

The design of school bags plays a crucial role in the weight distribution experienced by students, which can significantly impact their posture and overall physical health. Various bag styles, such as backpacks, messenger bags, and rolling bags, distribute weight differently, influencing how students carry their loads. Research indicates that poorly designed bags that fail to distribute weight evenly can lead to musculoskeletal pain, as heavily loaded one-shoulder styles can create imbalances that strain the spine and shoulders [5]. Furthermore, a heavy burden increases the risk of developing chronic postural issues during formative years, as children are still developing their musculoskeletal systems. Consequently, ergonomic bag designs that prioritize weight distribution are essential not only for comfort but also for preventing long-term health issues [6]. Teachers and parents play an essential role in educating students about the appropriate choice of bags to reduce these risks effectively.

The two-strap backpack is the most ergonomic design. One-strapped and hand-held bags may cause stress and strain on the back muscles due to an imbalanced load distribution between the shoulders. Roller bags also contribute to low back pain due to improper posture changes during their use. Carrying a backpack on one shoulder instead of both shoulders also increases the risk of low back pain due to postural deviation. Similarly, asymmetrical carriage contributes to low back pain as well [7].

The role of teachers and parents in guiding students on posture

The collaborative role of teachers and parents is crucial in guiding students on proper posture and school bag policies, which directly impact children's health and well-being. Teachers are positioned to observe student habits and intervene when improper carrying methods or postural issues arise, emphasizing the importance of using ergonomic school bags customized to their needs. Moreover, educational programs can be implemented within schools to raise awareness about the potential musculoskeletal pain linked to heavy school bags and improper posture; 78.99% of students reported experiencing pain, particularly in the shoulders and neck, due to excessive weight carried daily [8]. Meanwhile, parents play a pivotal part by monitoring their children's bag weight at home and reinforcing proper carrying techniques. This collective approach not only helps reducing the physical strain associated with heavy school bags but also fosters a culture of mindfulness towards health practices among students, thereby enhancing their educational experience and overall quality of life [9].

The posture of school children, as reported by parents, plays an essential role in understanding the implications of school bag design and weight on musculoskeletal health. Poor posture observed at home can serve as an early indicator of potential complications arising from the strain of heavy school bags. For instance, a retrospective study underscored that nearly 79% of students experienced musculoskeletal pain attributed to their school bag carriage, with significant reported discomfort in the shoulders and neck, closely linked to improper carrying methods and excessive weight [8]. This suggests that parents might not only recognize problematic postures at home but also be instrumental in advocating for ergonomic bag designs and policies within the school environment. Furthermore, the involvement of teachers in addition with parents is vital, as consistent guidance on proper posture can help establish healthier habits that can reduce the risks associated with inadequate bag policies.

Role of teachers

The role of teachers is pivotal in shaping student awareness and practices related to posture and bag-carrying methods, directly impacting their health. Educators serve as primary role models and are positioned to educate students about the physical implications of improper posture and heavy backpacks. By integrating discussions on health and body mechanics into the curriculum, teachers can foster an understanding of the importance of ergonomic practices. In a study, it is concluded that 80.2% children in private schools and 69.7% in government schools were carrying heavy school bags [10]. They can implement programs that encourage proper bag policies and teach students strategies for carrying their bags safely. Such proactive measures are essential, given that studies have indicated a correlation between heavy backpacks and musculoskeletal discomfort in adolescents [11]. Moreover, teachers can promote a supportive environment where students feel empowered to adjust their habits, thus bridging the connection between education and health promotion. This multifaceted approach not only improves student's immediate well-being but also inculcates lifelong healthy practices. Considering the gravity of the matter, children should be educated on ergonomics as a part of their school program, including instructions on carrying a backpack and the effects of disregarding the basic rules on body posture. The acceptable backpack load, which is currently believed to be 10% of the child's body mass, should be carefully considered [12]. A study's results revealed the relevant clinical features and risk factors, which help in the ergonomic recommendations and control measures that must be given by the school authorities/governing bodies to prevent or manage the incidents of backpack-related injuries among Indian school children. Also, it will help in the future for developing a comprehensive backpack injury prevention and management strategy [13].

Role of parents

The role of parents in guiding students on posture, bag-carrying methods, and adherence to the bag policy is pivotal for fostering lifelong healthy habits. Parents serve as primary role models, demonstrating proper body mechanics, which children often follow. For instance, when parents actively engage in discussions about the importance of ergonomic bag design and the appropriate ways to carry them, they instill awareness that can significantly impact their child's posture and overall health. Research indicates that parental involvement can enhance the effectiveness of educational programs, suggesting that parents who reinforce these principles at home help solidify the lessons learned in school. Moreover, creating an environment where schools and families collaborate in promoting healthful practices reinforces positive behaviors. Thus, the proactive engagement of parents not only supports the educational efforts of teachers but also encourages children to adopt healthier lifestyles early on, which can lead to sustained well-being.

Parental guidance helps in shaping students' habits related to posture and bag management, significantly impacting their overall health. When parents actively encourage good posture practices and proper bag-carrying techniques, children are more likely to adopt these health-promoting behaviors. This is evidenced by research indicating that parental influence is a strong predictor of physical activity and associated behaviors among adolescents, highlighting the importance of family engagement in fostering healthy habits [14]. Moreover, with the increasing use of technology, sedentary lifestyles are becoming common; thus, parents are essential in mitigating these trends, as they can model ergonomic practices at home and educate their children about the risks of poor posture linked to prolonged computer use [3]. Therefore, the collaborative encouragement from parents can lead to healthier habits in bag management and posture, which are essential for the prevention of musculoskeletal issues in students.

Strategies teachers can implement to educate students on proper posture and bag-carrying techniques

The responsibility of teachers in promoting proper posture and bag-carrying techniques extends beyond mere instruction; it involves the implementation of comprehensive educational strategies that engage students actively. One effective method is integrating posture awareness into existing physical education curricula, ensuring that students receive both theoretical knowledge and practical exercises that reinforce good habits. Workshops or seminars led by health professionals can provide additional insight, reinforcing the importance of posture in everyday activities and the health implications of improper bag-carrying techniques. Furthermore, peer-led initiatives can foster a supportive environment where students encourage one another to adopt healthy practices. By cultivating a classroom culture that values physical well-being, teachers can significantly impact students' habits and attitudes toward health, aligning with the aim of a favorable environment for creativity and innovation in learning, as articulated in this multifaceted approach, which plays a crucial role in shaping students' long-term health outcomes, supporting the essential cooperation among teachers and parents.

The design and weight of school bags play a critical role in influencing children's posture, an aspect often overlooked in educational settings. An improperly designed bag can shift a child's center of gravity, resulting in compensatory postural adjustments that lead to musculoskeletal discomfort over time. Research indicates that over 77% of adolescents report musculoskeletal pain, often exacerbated by poor ergonomic practices, such as carrying heavy or ill-fitted school bags [3]. Moreover, studies highlight that posture can deteriorate with prolonged use of laptops and mobile devices without proper support, suggesting that the weight and design of the bag directly affect how children position their bodies during academic activities [15]. Therefore, ensuring that school bags are lightweight and ergonomically designed is essential not only for improving children's physical comfort but also for promoting better long-term postural health as they engage in their studies.

The correlation between school bag design and children's posture is a critical consideration for educators and parents alike. Poorly designed bags, particularly those that are excessively heavy or lack appropriate ergonomic features, can facilitate improper posture, leading to musculoskeletal issues [16]. Research indicates that the weight distribution of school bags significantly impacts how children carry them, potentially resulting in slouched shoulders or misaligned spines [17]. Furthermore, specific design attributes, such as padded straps and back support, are essential in promoting better posture among school-aged children. Without these, the risk of developing chronic pain or discomfort increases, which can hinder academic performance and overall well-being. Addressing these design flaws is imperative to fostering a healthier environment for students and should prompt schools and manufacturers to prioritize ergonomically sound solutions in their bag designs. Ultimately, a well-designed school bag is not merely a convenience; it is a necessity for maintaining proper posture and supporting children's health.

The weight of school bags has significant implications for children's posture, as excessive load can lead to detrimental physical outcomes. Research suggests that a high percentage of school children carry bags that exceed the recommended weight limits, with some studies indicating that 40.1% perceive their bags as heavy [18]. Another research indicates that the extra weight of schoolbags results in back pain reported by 42% of school children; the recommended weight of schoolbags is less than 10-15% of the body weight [19]. Carrying such loads can disrupt natural body mechanics, leading to various musculoskeletal issues, particularly in the back and shoulders. Furthermore, another study highlights the relationship between physical activity levels and posture during walking with school bags; inactive children display more pronounced alterations in gait, revealing that they exert greater force on their feet compared to their more active peers [20]. These findings underscore the connection between bag weight and posture, emphasizing the need for careful consideration in bag design and weight management to promote better postural health in school children.

The increasing weight of school bags has emerged as a significant concern among parents and educators, particularly regarding its impact on children's posture. Several studies have reported a direct correlation between heavier bag weights and a rise in posture-related issues among school children, suggesting that overweight backpacks contribute to the development of musculoskeletal problems. For instance, the incidence and prevalence of low back pain in adolescents, as identified by [17], underscores the potential risks involved with 36% reporting low back pain in a 12-month period, a pattern that indicates the necessity for addressing bag weight. Furthermore, research highlighted in Harden et al. [21] suggests that inadequate bag design may exacerbate these issues, complicating the problem further. Thus, a holistic approach encompassing bag weight regulation and ergonomic design is essential in mitigating posture issues and promoting better health outcomes for school-aged children. Addressing these concerns is vital for fostering an environment conducive to optimal physical development.

Effects on student's/child's posture-related to heavy backpack

Students carry their bags in an unplanned manner, which may increase the weight of their school bags. Heavy school bags can have adverse effects on children's musculoskeletal systems, including headaches, muscle strain, and chronic back, neck, and shoulder pain.

A study found significant flattening of thoracic kyphosis when the backpack was worn on the participant’s right shoulder. There was a tendency for deepening of cervical lordosis, one of the most common sagittal plane changes in children has been the flattening of thoracic kyphosis, a forward bending of the head due to carrying the backpack asymmetrically, which increases both of these changes, irrespective of whether the backpack is carried on the left or right shoulder.

Carrying a backpack weighing 15% of body weight can change all the postural angles in preadolescent children. Students carrying heavier backpacks relative to their body weight are more likely to report back pain; the duration of use was strongly associated with both reported pain and severity [22].

The backpack load carriage impacts the gait of school children, resulting in significant changes to gait parameters. The physiological impacts of school backpack load on the students' respiratory muscle strength (inspiration and expiration) and lung function were reported in a study, which found that when students walk with more than 10% body weight load, there was an increase in breathing rate and a decrease in trunk range of movement. The physical discomfort caused by school backpack loads also includes bodily pain, redness, swelling, fatigue and/or musculoskeletal discomfort in the upper or lower back, upper or lower trapezius, shoulders, neck or forearms, with most of the students relating their pain, fatigue, and discomfort. The prevalence of back pain was reported to be higher among female students.

In a study, it was found that younger students (six to eight years) were more likely to carry heavier schoolbags than older students. Similarly, more girls than boys carried heavier bags, and girls were twice more likely to experience fatigue symptoms when carrying schoolbags compared with boys [23].

Discussion

Collective influence of teachers and parents on student health related to posture and bag policies: The interplay between teachers, parents, and peers significantly shapes student health, particularly regarding posture and bag policies. Teachers play a crucial role by integrating posture education into their curriculum, promoting awareness of healthy carrying techniques during classroom activities. Concurrently, parent's involvement in reinforcing these lessons at home, such as ensuring children carry suitable backpacks and encouraging regular breaks, fosters positive habits. Peers also contribute to this dynamic, as social interactions often drive conformity to acceptable behaviors, such as proper bag carrying and adherence to school regulations. Thus, the combined efforts of these three groups cultivate an environment that prioritizes student well-being, mitigating health issues associated with poor posture and heavy bag loads. This collaborative approach not only enhances academic performance but also establishes lifelong practices for physical health, emphasizing the importance of a united front in addressing these vital issues [24].

Schools should implement strategies to minimize the weight of students' bags and streamline academic practices, such as:·(1) Daily bag repacking and necessity: Encourage students to repack their bags daily, focusing on bringing only necessary items, such as articles, workbooks, and textbooks. Unnecessary items should be avoided; (2) Regular bag weight checks: Schools must ensure the weight of students' bags is regularly checked, especially for lower-grade students who often carry all their books to school; (3) Homework and bag policy for lower grades: For students in lower classes, schools should eliminate the assignment of homework or take-home assignments, and therefore, there should be no requirement for the students to bring school bags; (4) In-school homework completion: A separate timetable should be provisioned for students to complete their regular homework and assignments during school hours, under the direct guidance of teachers; (5) Locker provision: Schools should provide lockers for students to store items used at school, reducing the need to carry them back and forth daily.

Teachers help in alleviating the burden of heavy school bags and enhance the learning experience, schools should consider the following initiatives: (1) Embracing technology for learning: Teachers should actively explore and implement alternative teaching methods that leverage Information and Communication Technology (ICT). This approach can significantly reduce reliance on physical textbooks and workbooks. Examples include using digital learning platforms, educational apps, interactive whiteboards, and online resources for lessons and assignments; (2) Promoting resource sharing: A practical way to lighten bags is to encourage textbook sharing. Teachers are advised to pair students so that two children can share one textbook. This not only reduces the number of books each student carries but also promotes collaboration; (3) Minimizing paper load: Students should be encouraged to use loose sheets for their work purposes. This simple change can make a noticeable difference in bag weight compared to carrying multiple notebooks. Furthermore, teachers should also aim to carry fewer test copies, utilizing digital alternatives for grading and record-keeping where possible.

Parents play a crucial role in ensuring their children's well-being, particularly concerning the weight and proper use of school bags. Here's essential advice for parents: (1) Prioritize lightweight bags: The primary recommendation for parents is to prioritize the purchase of lightweight school bags to protect their children's health and prevent undue strain on their developing bodies; (2) Regular bag checks for unnecessary items: Parents should regularly check their child's school bag to identify and remove any unnecessary items. This consistent habit helps to prevent the accumulation of extra weight from articles, toys, or non-essential books; (3) Proper bag fit and wearing: Ensure the school bag is worn tightly to the child's back, with both shoulder straps utilized. This distributes the weight evenly and prevents the bag from hanging loosely off one shoulder, which can lead to poor posture and discomfort.

Implications for future school bag policies and posture education

This study's findings underscore the critical relationship between school bag design, weight, and the reported posture of students, revealing that improperly designed and excessively heavy bags contribute significantly to postural issues in schoolchildren [12]. It is evident that both parents and teachers play an important role in guiding students toward better posture and adopting effective bag usage policies. In light of these results, future school bag policies should advocate for lighter, ergonomically designed backpacks that can be adjusted to fit each child's physique, thereby alleviating undue strain on their developing bodies. Additionally, incorporating posture education into school curriculums would empower children to recognize and correct poor posture habits, fostering a culture of health and wellness. The collaboration between educators and parents in monitoring and instructing children on proper posture and bag handling can lead to significant improvements in student's overall health and academic performance.

To ensure ease in carrying school bags by students, particularly in varying distances to school and weather conditions, here are the essential features to consider: (1) Lightweight: The bag itself should be light to avoid adding extra burden to the child's back and shoulders; (2) Padded and adjustable shoulder straps: Look for straps that are well-padded for comfort and can be adjusted to fit the child's body properly, ensuring the bag sits at the correct height; (3) Multiple compartments: These help in organizing items, distributing weight evenly, and reducing clutter; (4) Sternum strap: This strap helps to spread the bag's weight across the chest, stabilizes the bag and helps distribute weight hence promoting better posture; (5) Hip belt: A hip belt is beneficial as it shifts some of the weight from the shoulders to the stronger hip muscles, this should also be adjustable to effectively transfer weight from the shoulders to hips; (6) Contoured back panel: A back panel that is ergonomically shaped to follow the natural curve of the spine is vital. This design helps in promoting good posture aligning with the natural spinal curve rather than pulling it out of alignment and reducing strain by distributing the weight more evenly across the entire back surface; (7) Breathable materials (mesh panels): In a warm and often humid climate, it can improve ventilation, particularly with mesh panels on the back panel and the underside of shoulder straps, allows for air circulation and reduced moisture buildup. This prevents heat and sweat from accumulating between the student's back and the bag, significantly enhancing comfort, preventing chafing, and reducing odors; (8) Smart weight distribution (integrated design): This refers to the overall design approach of the bag to ensure weight is handled optimally by even load spreading, minimizing strain and strategic placement; (9) Adjustable compartments (internal dividers/straps): While "multiple compartments" are important, "adjustable compartments" offer greater flexibility; such features allow students to customize the internal space based on the size and type of items they are carrying on a given day (e.g., larger textbooks one day, sports gear another). This helps secure items and prevents them from shifting, which can throw off balance; (10) Reinforced stitching and durable materials: These features directly impact the bag's longevity and reliability, which is crucial for daily school use.

Evaluating the use of rolling bags, there are several key factors that should be taken into account to ensure their practicality, safety, and effectiveness. The versatility can be achieved through several innovative design approaches, such as convertible rolling bag designs, which help to offer the best of both, the ease of rolling heavy loads and the comfort of carrying a backpack when wheels are impractical. These considerations can be broadly categorized as follows: (1) Storage and organization: Rolling bags should offer sufficient space to accommodate all necessary belongings, including books, notebooks, laptops, and personal items; (2) Designated rolling bag-friendly areas: Schools should clearly identify and communicate areas where rolling bags can be used safely. This typically includes flat, unobstructed surfaces, wide corridors, and designated storage zones. Consider the presence and accessibility of stairwells and ramps; (3) Hybrid bag design (seamless integration): This design seeks to blend the features of a rolling bag and a backpack. It's often a refinement of the retractable handle or highly integrated detachable wheel designs. The retractable handle can be fully retracted and concealed within a dedicated zippered compartment or sleeve when not in use. When the handle is retracted, the bag can be unzipped or adjusted to reveal and deploy the backpack straps. The back panel should be designed to either cover the handle mechanism or incorporate sufficient padding to make it comfortable against the user's back. Also, detachable/foldable wheels can be easily removed or folded inside the main bag structure. Once the wheels are removed, the bag can be worn as a backpack.

Ergonomic bags offer several biomechanical advantages, which contribute to reducing physical strain and enhancing comfort during load carriage [6]: (1) Reduced shift in center of gravity: Ergonomic bags are designed to keep the load close to the body, minimizing the shift in the center of gravity. This helps maintain balance and reduces the strain on muscles and joints; (2) Improved biomechanics: By promoting natural movement patterns, ergonomic bags help reduce abnormal stress on the musculoskeletal system, lowering the risk of discomfort and injury; (3) Even weight distribution: These bags evenly distribute weight across both shoulders or the back, minimizing localized pressure and reducing the chance of muscle fatigue; (4) Better posture: The supportive structure of ergonomic bags encourages users to maintain an upright posture, thereby reducing strain on the spine and associated muscles; (5) Reduced muscle fatigue: Even weight distribution and posture support help prevent the early onset of muscle fatigue during prolonged use; (6) Improved movement patterns: Ergonomic designs allow for more natural walking and body movement, reducing compensatory motion and stress; (7) Enhanced comfort: Features like padded straps, lumbar support, and secure fit improve comfort and reduce pressure points.

Awareness should be created among health care professionals, teachers, and parents to restrict backpack load less than 5% of bodyweight by using school locker shelves, compact discs, and USB flash drives and need to regularly monitor the musculoskeletal problems associated with carrying heavy backpack load in preadolescent children. So musculoskeletal dysfunction and its relation to preadolescent postural responses to backpack load need to be further explored through longitudinal and prospective studies, respectively, to determine whether carrying a backpack increases the incidence of regional pain and to correlate these clinical implications on school children.

In a study, there were differences found in experiencing musculoskeletal pain in Indian school children and American school children of the same age group; the Indian students are younger, lower in height, BMI, arm and shoulder strengths, and body fat, compared to American school children [25]. Interestingly, the Indian students carry more to school, but the American students perceive more pain than Indian school children.

We have also incorporated key conclusions from academic studies to substantiate the issues children face due to heavy backpack loads (Table 1).

**Table 1:** Key conclusions from academic studies to substantiate the issues children face due to heavy backpack loads.

S. no	Article	Author and year	Study findings	Ref. no.
1	Musculoskeletal pain and backpack usage among School children in Nairobi County, Kenya	Ogana et al. (2017)	Musculoskeletal pain is significantly associated with school bag weight, pupil's weight and bag to school child's weight percentage. School children who carry backpacks that weigh more than 15% of their body weight are at risk of experiencing musculoskeletal pain.	[2]
2	School bag usage, postural and behavioral habits and its effect on back pain occurrence among school children	El-Nagar et al. (2017)	There was a significant relationship between back pain and school bag weight, % of body weight, method and duration of carriage.	[25]
3	Effect of bagpack weight on postural angles in preadolescent children	Ramprasad et al. (2010)	Carrying a backpack weighing 15% of body weight changes all the postural angles in preadolescent children.	[11]
4	Low back pain in schoolchildren: the role of school bag weight and carrying way	Farhood (2013)	In this study, lower back pain was associated with heavy school bag as 56.7% and 35.1% had LBP and neck-shoulder pain, respectively.	[18]
5	A study of school bag weight and back pain among primary school children in Al-Ahsa, Saudi Arabia	Al-Saleem et al. (2016)	Back pain was reported by 42% of the school children due to heavy school bags.	[19]
6	The impact of backpack loads on school children: a critical narrative review	Perrone et al. (2018)	Study shows that the impacts of heavy load of bags may include changes to posture (e.g., changes to spinal posture, lumbo-sacral angles, and thoracic kyphosis), gait (increases in plantar pressure during foot-ground contact and increased double support), increases in physical discomfort and muscle activity, and increases in breathing rate.	[24]
7	Acute stress and strain due to backpack loading among primary school pupils.	Abrahams (2011)	The excessive schoolbag mass carried by the students of primary sections placed strain on the immature vertebral column of thus causing postural deviations which induced musculoskeletal pain and discomfort.	[8]
8	The effects of age and gender on the weight and use of schoolbags	Kellis et al. (2010)	Younger children are in greater need for education about schoolbag weight compared with older ones. There is also evidence that girls might experience more problems when carrying their schoolbag compared with boys.	[23]
9	Effect of asymmetrical backpack load on spinal curvature in school children	Drzał-Grabiec et al. (2015)	The asymmetrical backpack load resulted in a significant reduction of thoracic kyphosis, children should be educated on ergonomics by teachers, physiotherapists, or nurses, including instructions on carrying a backpack and the effects of disregarding the basic rules on body posture, as part of their school curriculum.	[12]
10	Impact of educational intervention in reducing the weight of school bags in selected school children	Saini et al. (2023)	Children carrying heavy bags were suffering from musculoskeletal problems. Children carrying heavy bags reported musculoskeletal problems five times higher than their counterparts.	[10]
11	Backpack carriage and musculoskeletal pain among primary school learners in King William's Town, South Africa	Moni (2022)	Most schoolchildren suffer from musculoskeletal pain because the weight of their backpacks is excessive compared to their size, weight, and age.	[9]

## Conclusions

In summary, the evidence presented underscores a clear relationship between the design and weight of school bags and the postural health of children. The findings from the study indicate that ergonomic backpacks with additional straps can significantly improve posture among schoolchildren, as illustrated by changes in the craniovertebral and cranio-horizontal angles and sagittal shoulder posture after their use. Conversely, the prevalence of musculoskeletal pain linked to the use of heavy backpacks highlights the urgent need for intervention. A significant proportion of students reported discomfort, particularly in their shoulders and lower back, attributed to carrying bags that exceed recommended weight limits. These findings compel the adoption of better bag designs and educational programs for stakeholders, including parents and teachers, to promote healthier carrying practices and thereby foster improved posture and overall well-being among school-aged children. The evidence supports that both school bag design and weight significantly influence children's posture.

Parent-reported outcomes align with clinical assessments, indicating that improper bag characteristics contribute to musculoskeletal strain. Interventions such as limiting bag weight, promoting ergonomic designs, and educating children and parents on proper carrying techniques could mitigate postural issues. The association between school bag design and weight has significant implications for the well-being of school children, prompting critical considerations for parents, educators, and manufacturers alike. Findings from this study suggest that improperly designed bags contribute to poor posture, which can lead to long-term health issues. Parents should be proactive in choosing ergonomic bags that distribute weight evenly and encourage their children to carry only necessary materials. Educators can play a vital role by instituting guidelines for acceptable bag weight and design, fostering an environment that prioritizes student health. Furthermore, manufacturers are called upon to innovate and create versatile, lightweight materials that also cater to the aesthetic preferences of students. Collective action from these stakeholders can mitigate the adverse effects of heavy backpacks, promote healthier postural habits, and support the overall academic experience. Ultimately, a collaborative approach will nurture both the physical and educational development of school-aged children.
